# Move it or lose it: Predicted effects of culverts and population density on Mojave desert tortoise (*Gopherus agassizii*) connectivity

**DOI:** 10.1371/journal.pone.0286820

**Published:** 2023-09-28

**Authors:** Kirsten E. Dutcher, Kenneth E. Nussear, Jill S. Heaton, Todd C. Esque, Amy G. Vandergast

**Affiliations:** 1 Department of Geography, University of Nevada–Reno, Reno, Nevada, United States of America; 2 United States Geological Survey, Western Ecological Research Center, Boulder City, Nevada, United States of America; 3 United States Geological Survey, Western Ecological Research Center, San Diego, California, United States of America; Central University of Punjab, INDIA

## Abstract

Roadways and railways can reduce wildlife movements across landscapes, negatively impacting population connectivity. Connectivity may be improved by structures that allow safe passage across linear barriers, but connectivity could be adversely influenced by low population densities. The Mojave desert tortoise is threatened by habitat loss, fragmentation, and population declines. The tortoise continues to decline as disturbance increases across the Mojave Desert in the southwestern United States. While underground crossing structures, like hydrological culverts, have begun receiving attention, population density has not been considered in tortoise connectivity. Our work asks a novel question: How do culverts and population density affect connectivity and potentially drive genetic and demographic patterns? To explore the role of culverts and population density, we used agent-based spatially explicit forward-in-time simulations of gene flow. We constructed resistance surfaces with a range of barriers to movement and representative of tortoise habitat with anthropogenic disturbance. We predicted connectivity under variable population densities. Simulations were run for 200 non-overlapping generations (3400 years) with 30 replicates using 20 microsatellite loci. We evaluated population genetic structure and diversity and found that culverts would not entirely negate the effects of linear barriers, but gene flow improved. Our results also indicated that density is important for connectivity. Low densities resulted in declines regardless of the landscape barrier scenario (> 75% population census size, > 97% effective population size). Results from our simulation using current anthropogenic disturbance predicted decreased population connectivity over time. Genetic and demographic effects were detectable within five generations (85 years) following disturbance with estimated losses in effective population size of 69%. The pronounced declines in effective population size indicate this could be a useful monitoring metric. We suggest management strategies that improve connectivity, such as roadside fencing tied to culverts, conservation areas in a connected network, and development restricted to disturbed areas.

## Introduction

Connectivity can be defined as the ability of a landscape to support ecological and evolutionary processes such as animal movements, gene flow, and range shifts [1]. The degree to which a landscape maintains connectivity affects population sizes and genetic structure across a broad range of taxa [2–5]. Studies have found that as individuals lose the ability to move across their former range population size decreases, genetic diversity decreases and genetic differentiation increases [6–8]. Complex interactions between demographic characteristics, such as density, and landscape characteristics, such as habitat amount and configuration [9, 10], can alter rates of dispersal and gene flow between populations [11]. The ability of low density populations to maintain connectivity has not been explicitly explored. Hypothetically, connectivity may be reduced if population density is low and individuals are unable to find mates [12]. This could produce small populations that may experience genetic drift and lose genetic diversity [6, 13, 14], which is a fundamental component of biodiversity and provides raw material for adaptation and evolution [15, 16].

Our focal species, the Mojave desert tortoise (*Gopherus agassizii*) is federally listed as threatened because of declines in population density and habitat [17]. Range-wide population density monitoring has found that tortoise densities vary across the landscape, are related to habitat, and have been negatively impacted by habitat loss and degradation [18]. Since their listing, desert tortoises have continued to decline [17, 19–21]. Estimates of an approximately 37% loss of adults range-wide from 2004 to 2014 have been reported [18]. Ongoing recovery actions related to increasing density include installation of tortoise barrier fencing, protecting and connecting habitat within and among conservation areas, and targeted population augmentation [17]. However, the effects of population density on Mojave desert tortoise connectivity have not been considered. Anthropogenic linear features, like roadways and railways modify behavior and restrict movement [22], with emergent genetic consequences in multiple species [23, 24]. Roads have been found to alter and reduce tortoise movements [25, 26], resulting in a genetic signal of fragmentation [27]. Roads also cause direct mortality when unfenced [28] and reduce relative abundance within several kilometers of roadways [29–31]. Reduced abundance of tortoise sign is correlated with habitat degradation along roadsides, with impacts extending farther from roads themselves as traffic load increases [32].

When a natural landscape becomes fragmented, road crossing structures and other corridors may maintain or even restore connectivity [33]. Functional corridors can enhance population viability by allowing individuals to move safely across the landscape [34–36]. Gopher tortoises (*Gopherus polyphemus*) will cross railroad tracks using trenches dug underneath the rails, improving demographic connectivity [37]. In the Mojave Desert, hydrological culverts along linear barriers are placed in washes, which tortoises often use as movement paths [26]. In semi-natural experiments, Mojave desert tortoises walked hardwire mesh fences and entered culverts under a large highway (Interstate 15) [38]. However, many culverts are currently inaccessible due to roadside fencing. Therefore, evaluation of the effectiveness of underground crossing structures, such as culverts, in maintaining population connectivity is needed to guide management decisions [39] concerning culvert accessibility.

There are multiple ways to evaluate connectivity, including demographic (the impact of dispersal on population growth rates) and genetic (evolutionary consequences of dispersal or gene flow) [40]. Dispersal movements are poorly understood in the desert tortoise. Tortoises are believed to reside in habitat areas with average home-range estimates of 1 km^2^ and have limited dispersal ability [25, 41]. In species with limited dispersal, genetic tools can provide a framework to examine hard-to-observe processes like movement across a landscape [40, 42–45]. Incorporating genetics with demographic metrics, such as population size, can improve our estimates of connectivity across a landscape [17, 40].

Understanding landscape spatial structure is central in evaluating connectivity because landscape amount and configuration influence population size and genetic structure [24, 46, 47]. The use of spatially explicit simulations can enhance our understanding of the role landscape plays in shaping population size and genetic structure [48–51]. Spatial agent-based models add realism by explicitly incorporating landscape, allowing for the study of land use changes on the structure of populations. The cost of movement, or ability of an individual to move through its environment, can be represented using a landscape resistance surface [52]. Resistance values (i.e. high resistance may be assigned to urban areas or major roads) for each pixel cell in a gridded raster allow movement to be modeled as a function of features on a map [43].

The lag time associated with detection of genetic patterns following changes on the landscape can make empirical evaluations of gene flow in relation to contemporary disturbance challenging. This is exacerbated by long generation times [39]. Limited dispersal ability has also been found to increase the lag time for detecting genetic differentiation (*F*_*ST*_) associated with landscape barriers (up to 200 generations) [53]. Because desert tortoises have limited dispersal ability and long generation times [54], it would likely require decades, or longer, to empirically determine the genetic effects of fragmentation from recent linear barriers, the potential connective value of underground culverts, and the consequences of population density. However, forward-in-time simulations can aid in evaluating how changing landscape features and declining population densities are predicted to influence future populations [55–57].

Our work asks a novel question in the field of landscape genetics: How do culverts and population densities affect connectivity and potentially drive long-term genetic and demographic patterns? This study used agent-based models to simulate gene flow forward-in-time to better understand tortoise connectivity and population size in four landscapes (three hypothetical and one real): without a linear barrier, with an absolute barrier to movement, with the addition of connectivity culverts along the linear barrier, and in a heterogeneous landscape with multiple complex features. Simulations using hypothetical landscapes were run at variable initial population densities. We tested the following hypotheses: 1) the addition of culverts across roads and railways will maintain or improve gene flow for desert tortoises and 2) reduced population density in the surrounding landscape will negatively influence connectivity. We applied the simulation framework to a real world study landscape that may represent an important population linkage for wild desert tortoises [58, 59]. However, rapid and recent anthropogenic disturbance in the area may reduce population density and disrupt natural patterns of gene flow. Modeling the impacts of crossing structures and population density could provide managers with valuable information to weigh the benefits of investing in improving local population densities and culvert access for desert tortoises.

## Methods

### Simulation model

We performed agent-based spatially explicit genetic simulations of gene flow for 200 generations (approximately 3400 years), using the program SimAdapt v.1.8.0 [60], with 30 replicated runs, sampling individuals every five generations (roughly every 85 years). SimAdapt uses the NetLogo environment [61] to model mating and dispersal in non-overlapping generations within a georeferenced area with closed boundaries. The simulation modeling platform allows density to vary across the landscape and tracks individuals through time. The program simulates landscape genetic processes with user defined parameters, including initial genetic structure, and records the alleles of all individuals from forward-in-time generations [56]. This allowed us to explore variations in population density and genetic signals to test the influence of barriers and systematically evaluate genetic and demographic connectivity on variable landscapes. Below and in Table 1 we describe the specific model inputs and parameters.

**Table 1:** Parameters of the simulation models.

Parameters	Value/Description	Justification
Landscape Extent	25 x 25 km (625 km^2^)	Area is approximately the size of Ivanpah Valley genetic cluster
Landscape Scenarios	no barrier, barrier, culverts, real world study area	Allow for comparison
Grid Cell Size	1 km^2^	[25, 41, 62]
Cell Carrying Capacity	3, 14, 24, variable tortoises per cell	[18]; 1 km^2^ study plots
Initial Population Size	1875, 8750, 12500, 7025	Defined by density and landscape
Dispersal Probability	0.5	Hromada pers comm 2019
Dispersal Distance	≤ 10 cells approximately 14 km	[63]
Population Growth Rate	0.48	[54, 64]
Offspring Sex Ratio	1:1	[65]

### Barrier resistance landscapes

**Fig 1:**
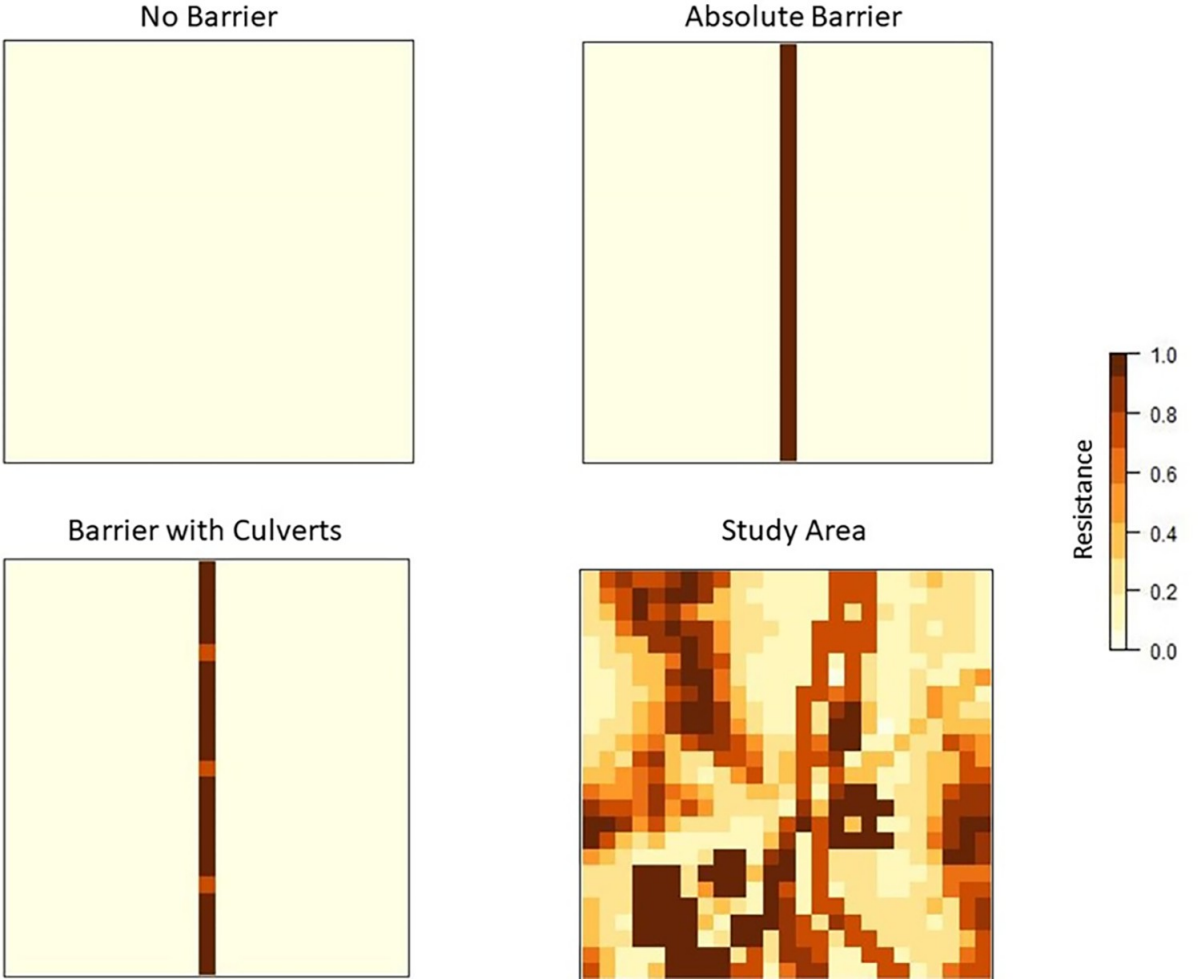
Landscape resistance surfaces. Three hypothetical landscapes (no barrier, absolute barrier that is one grid cell wide, barrier with culverts) and a real world study area in the Ivanpah Valley along the Nevada/California border were created. The Ivanpah Valley surface used the inverse of desert tortoise habitat suitability [62], with the addition of existing anthropogenic disturbance layers. Disturbance was applied using conversion factors to adjust cell values and increase resistance. Types of disturbance are reported in Table 2. The extent of each surface is 25 x 25 km with a resolution of 1 km^2^.

We characterized landscapes using resistance surfaces constructed and mapped in R 3.5.3 [66] using packages *ggmap* v.3.0.0.901, *raster* v.2.9–5, and *rgeos* v.0.4–3 [67–69]. In preliminary work, we found that the simulated landscape size was sufficient to allow isolation-by-distance (IBD) to form. At larger spatial extents the increased number of individuals resulted in intermittent failure due to limitations with computing genotypes. Resistance values represent the cost of movement across a gridded landscape where ease of movement ranges from no resistance (0) to no movement (1). We created landscapes constructed as a uniform surface with no resistance, a surface with an absolute barrier to movement, and a surface with three culverts embedded within the linear barrier (Fig 1). These resistance surfaces allowed for evaluation of differences in genetic diversity, genetic structure, and simulated population size in simplified scenarios allowing for IBD. We tested specific landscape genetic hypotheses of connectivity across linear barriers and the potential influence of population density.

Barriers did not allow movement unless a culvert was present. We defined the culvert resistance value using the inverse of a desert tortoise habitat suitability model [62]. The inverse of habitat suitability has been assumed to be a valid proxy for resistance [52, 58] and may be appropriate for species that require occupancy within their movement areas. Simulated tortoises were present across the range of potential habitat values (0.1 to 1), but with a low probability of presence between 0.1 and 0.3 [62]. Because tortoise use of culverts has been documented [38], but culverts do not represent habitat, we assigned culverts a resistance value of 0.7 (habitat suitability value of 0.3), to allow for a low probability of use (three animals could occupy or move through that cell each generation).

### Ivanpah Valley resistance landscape

We created a landscape resistance surface of the Ivanpah Valley, situated along the Nevada/California border and considered central to range-wide connectivity for desert tortoises [58]. Cell values were determined using the inverse of desert tortoise habitat suitability [62].

Because habitat becomes less suitable with anthropogenic disturbance, we reduced the habitat suitability value of cells containing disturbance on our landscape resistance surface (Fig 1). Habitat suitability values were adjusted using conversion factors as numerical estimates of possible degradation. We applied conversion factors to cells with urban and solar disturbance, a railway and interstate, minor roads (secondary and dirt), and utility corridor right-of-ways (Table 2). We applied a relatively low maximum conversion factor to minor roads based on road density in a cell, so that cells with more dirt roads were associated with penalties at or below the maximum value. Following conversion, we calculated resistance by taking the inverse of the resulting values. Conversion factors were adapted for desert tortoises and calculated with the equation used by Inman et al. [70] as:

habitatsuitability–(habitatsuitability×conversionfactor)

**Table 2:** Conversion factors, habitat values, and resistance values.

Disturbance	Conversion Factor	Example Habitat Value	Adjusted Habitat Value	Resistance Value
None	0	0.500	0.500	0.500
Urban and Solar	1.00	0.500	0	1.000
Railway and Interstate	0.75	0.500	0.125	0.875
Minor Roads (max length)	0.25	0.500	0.375	0.625
Rights-of-Way	0.25	0.500	0.375	0.625

Conversion factors were used to adjust habitat suitability values for cells with anthropogenic disturbance. The inverse of desert tortoise habitat suitability [62] was used to calculate landscape resistance.

### Population density assignments

Each cell was assigned a maximum density based on the resistance value (see Table 1). Because empirical density estimates for adult desert tortoises are highly variable (0.2 to 28/km^2^) [18], we used estimates from 1 km^2^ study plots from ongoing research in Ivanpah Valley to define high density. We represented moderate and low densities based on the relationship between habitat suitability and population density. Individuals were assigned to geographic populations based on their location relative to the linear barrier (i.e. on either side of the road). Habitat in the hypothetical landscape scenarios (no barrier, absolute barrier, and barrier with culverts) was assigned no resistance to movement and modeled at three densities: high, moderate, and low. For the Ivanpah Valley simulation, the carrying capacity ranged from 0 (cells with maximum resistance) to 24 tortoises (cells with no resistance, Table 3).

**Table 3:** Carrying capacities.

Habitat Value	Resistance Value	Carrying Capacity
0	1.00	0
0.01–0.10	0.90–0.99	1
0.11–0.20	0.80–0.89	1
0.21–0.30	0.70–0.79	3
0.31–0.40	0.60–0.69	6
0.41–0.50	0.50–0.59	9
0.51–0.60	0.40–0.49	12
0.61–0.70	0.30–0.39	15
0.71–0.80	0.20–0.29	18
0.81–0.90	0.10–0.19	21
0.91–1.00	0–0.09	24

Carrying capacities in 1 km^2^ grid cells determined by habitat suitability values from a desert tortoise habitat suitability model [62]. Resistance values calculated as the inverse of the habitat value of each grid cell.

### Simulation parameters

**Fig 2:**
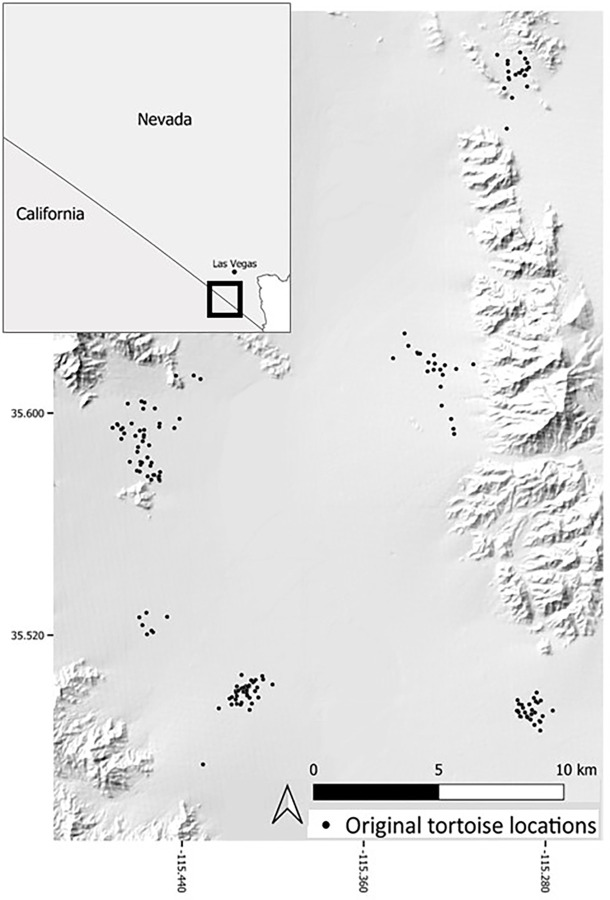
Map of tortoise locations. Individuals were from the Ivanpah Valley, along the Nevada/California border, and used as the original genetic data in forward-in-time simulations (*n* = 170). The state overview map has been adapted from the USGS National Boundary Dataset public domain and is for illustrative purposes only.

Because putatively neutral genetic markers like microsatellites are not influenced by selective forces, they are ideal for investigations of gene flow [71]. We simulated 20 variable microsatellite loci [72–74] from an empirical dataset using a subset of data from a previously identified genetic cluster in the Ivanpah Valley (Fig 2) [27, 75]. Detailed information on genotypes selected is available in S1 Table. We created genotypes for simulation scenarios by randomizing the original samples to remove any potential signal of IBD. We then generated a large genotype file (*N* = 14572) using our simulation framework on a uniform landscape surface with a burn-in of 100 generations. We compared the simulated data (*n* = 750) with the original data (*n* = 170). All loci conformed to Hardy-Weinberg equilibrium following Bonferroni correction (*p* < 0.003).

In our simulations, time was measured in generations as discrete steps. Non-overlapping generations is a simplification that allows patterns to be more clearly detected; however, it may underestimate time to detection [76]. In the simulations all adults died at the end of each time step. At the start of the next time step all surviving offspring became breeding adults. We defined a generation as the average length of time to reach reproductive maturity, estimated at 17 years [17, 77].

Individuals dispersed, reproduced, then died in that order. Individuals were selected at random for dispersal movements, reproduction, and death. As there are no empirical data on an individual’s dispersal likelihood, we used telemetry data from Ivanpah Valley to calculate the percent of animals that left 1 km^2^ study plots without returning to approximate the probability of dispersal (Hromada pers comm 2019). Simulated dispersal was not directional. To allow movement in any direction, including diagonally, individuals were allowed to move to one of eight neighboring cells (Queen’s case), then up to 10 cells (≤ 14 km) per generation [63]. Individuals traveled the maximum distance allowed, provided movement was possible on the landscape. The destination cell was chosen randomly among possible cells. There is evidence that tortoise movements are not random [25, 26, 41]. Tortoises also exhibit social behaviors related to burrow use that may influence mating behaviors [78]; however, how tortoises find mates is not known. In our simulations reproduction depended on finding mates within the same cell. Should no mate be available, individuals could not reproduce.

The number of surviving offspring was controlled by a logistic growth function using the number of individuals in a given cell, carrying capacity, and an intrinsic growth rate. For the percent population growth per generation we used a growth rate based on an annual growth estimate of 1% multiplied by 48 breeding years, based on average lifespan [54, 64] minus average age of reproductive maturity (17 years) [17, 77]. Transmission of alleles followed Mendelian laws of inheritance. No loci were under selection, and we used a mutation rate of 0.0005 per locus per generation [53, 76, 79]. This value falls within the range of mutation rates for microsatellite loci estimated by Edwards et al. [80] for desert tortoises. A detailed description of simulation methods is available in S1 Table.

### Analysis of simulation output

We examined simulated data through time using outcomes averaged across replicate simulations. We randomly sampled large datasets without replacement to create subsamples for analyses. To ensure our sample size was sufficiently large to capture genetic effects, we plotted pairwise *F*_*ST*_ values against sample sizes of 100–1200 that were taken from the moderate density simulation with no barrier at generation 200. We selected a sample size (*n* = 750) that fell after estimates of pairwise *F*_*ST*_ converged. Individuals were grouped based on geographic location relative to the linear barrier. We compared simulated population dynamics by evaluating the total number of individuals through time by landscape and population density scenario. Ivanpah Valley simulation results were compared with original genetic data (current Ivanpah Valley population genetic diversity and structure adapted from Dutcher et al. [27]).

To monitor genetic consequences, we calculated effective population size (*Ne*) at the landscape level and for each subpopulation on either side of a linear barrier. Effective population size estimates the size of an idealized population experiencing the same loss of diversity through drift as the population under study [81]. In our models with non-overlapping generations, *Ne* estimated from a single generation or cohort approximates the number of breeders that contributed to the cohort in which it was measured [82, 83]. Effective population sizes were estimated using the linkage disequilibrium method under a random mating model implemented in *NeEstimator* v.2.1 [84, 85]. We used 0.05 for the lowest allele frequency with 95% parametric confidence intervals.

We also measured genetic diversity as allelic richness (*Ar*), and predicted observed and expected heterozygosity (*H*_*o*_ and *H*_*e*_) at the landscape level and for each subpopulation on either side of a linear barrier. Genetic diversity statistics were calculated with the R package *adegenet* v.2.1.1 [86]. We investigated pairwise *F*_*ST*_ [87] between groups in the R package *hierfstat* v.0.04–22 [88] in time-series, using outcomes averaged across replicate runs, reporting the standard deviation. We selected genotype files best representing the mean *F*_*ST*_ for downstream analyses.

We examined population genetic structure at generation 200 with a Bayesian clustering analysis (Structure v.2.3.4) [89]. We used the admixture model to allow for mixed ancestry, correlated allele frequencies, and location as a prior to improve inference when genetic structure is weak. We estimated the probability of *K* population clusters (ranging from 1 to 10) using 10 replicate runs of 1,000,000 Markov Chain Monte-Carlo iterations following a burn-in of 500,000 iterates. Results were visualized using *PopHelper* v.2.2.9 in R [90]. In the presence of spatial autocorrelation, Structure may misrepresent genetic clusters [91, 92]. Because the mean log probability of the data (*Pr(X|K)* [89]) may overestimate clusters with IBD, we report the number of clusters based on the second order rate of change (Δ*K* [93]), except where *Pr(X|K)* = 1. Additionally, we used spatial principal components analysis (sPCA in R package *adegenet* v.2.1.1, [94] to evaluate cryptic genetic patterns in the presence of IBD. We analyzed generations five and 40 (approximately 85 and 680 years; respectively) because they represent standardized time periods for short and long-term persistence [95]. We also analyzed generation 200, the final generation simulated. sPCA is a multivariate method that maximizes genetic diversity (variance) in individual allele frequencies while accounting for spatial structure (spatial autocorrelation measured by Moran’s *I*). We compared the genetic patterns to 999 randomized Monte-Carlo permutations to test for differences between observed structure and the distribution of random expectations. Mantel tests were performed on sPCA data to determine the significance of the correlation between genetic and geographic distances. Because a Mantel test uses whole data and Moran’s I uses a portion of the data, the former may indicate stronger patterns while the latter may find more cryptic structure; therefore, we report *p*-values for both.

## Results

Each simulation scenario was seeded based on population density (see Table 1). There were no significant differences in *H*_*o*_ and *H*_*e*_ between the original dataset used to create individuals for simulation scenarios and the simulated individuals (*p* > 0.05, *df* = 19; Table 4).

**Table 4:** Genetic statistics for original data and initial simulations.

Genotypes	*n*	*Ne* (CI)	*Ar* (SD)	*H*_*o*_ (SD)	*H*_*e*_ (SD)	*F*_*ST*_
Original	170	218 (187–260)	13.0 (7.0)	0.80 (0.13)	0.81 (0.12)	0.002
Simulated (sample)	750	6853 (3410–163494)	21.0 (9.6)	0.79 (0.11)	0.82 (0.11)	0.001
Simulated (all)	14572	10256 (9188–11523)	26.2 (13.0)	0.79 (0.11)	0.82 (0.11)	0.001

Number of individuals (*n*) used in calculations of: (*Ne*) effective population size with 95% confidence intervals; (*Ar*) allelic richness; (*H*_*o*_) mean observed and (*H*_*e*_) expected heterozygosity; (*F*_*ST*_) overall genetic differentiation. Original tortoises are those sampled within the Ivanpah Valley following randomization. Simulated (all and sample) are the initial simulated individuals used in density, barrier, and Ivanpah Valley simulations.

### Barrier and density simulations

By the final simulated generation, the total number of individuals was highest with no resistance and lowest with an absolute barrier in all simulations. The absolute barrier represented a major road and decreased movement as well as occupancy. Reduced tortoise abundance has been documented well beyond the footprint of a major road and may reflect decreased habitat quality [29, 30]. Roads can also increase habitat loss and degradation by facilitating the spread of invasive plants, increasing soil erosion, and changing hydrologic patterns [96].

The total number of individuals in moderate and high density simulations remained stable through time with the no barrier landscape. However, in the absolute barrier landscape, and with the addition of three culverts along the 25 km barrier, the average number of individuals decreased by 8%. The population census size decreased by 75% and *Ne* decreased by 98% to 99.9% in low density simulations regardless of landscape because of lack of mates within the dispersal cell (Table 5). To further characterize the effect of a barrier we compared *Ne* in the barrier and culvert simulations with the no barrier simulation. In the low density scenarios, we predicted relative rates of decline in *Ne* of 80% (barrier) and 9% (culvert). Moderate and high density simulations had the most dramatic decreases in *Ne* in the barrier simulations (72% to 82%), with some improvement when culverts were present (61% to 66%), although losses were still high (Table 5). Relative rates of *Ne* decline in the moderate density scenarios were 55% (barrier) and 37% (culvert). In the high density scenarios, rates were estimated at 93% (barrier) and 86% (culverts). The reported estimates of relative declines in *Ne* may be steep due to wide confidence intervals at moderate and high densities (Table 5).

**Table 5:** Density and barrier simulation results from hypothetical landscape scenarios.

Density	Landscape	Level	*N* (SD)	*Ne* (CI)	*Ar* (SD)	*H*_*o*_ (SD)	*H*_*e*_ (SD)	*F*_*ST*_
Low	No Barrier	**Pop**	**460 (12.3)**	**160 (150–172)**	**14.2 (6.2)**	**0.31 (0.02)**	**0.79 (0.01)**	0.007
	Barrier	**Pop**	**434 (9.7)**	**32 (30–33)**	**11.9 (5.8)**	**0.29 (0.02)**	**0.79 (0.02)**	0.073
		Subpop	216 (6.4)	86 (78–95)	12.0 (5.5)	0.29 (0.02)	0.73 (0.03)	
			216 (6.2)	80 (74–88)	11.9 (5.5)	0.29 (0.02)	0.73 (0.02)	
	Culverts	**Pop**	**438 (8.1)**	**146 (136–157)**	**13.3 (5.9)**	**0.30 (0.02)**	**0.79 (0.01)**	0.020
		Subpop	219 (9.0)	105 (95–115)	13.4 (6.0)	0.31 (0.02)	0.77 (0.01)	
			217 (11.1	87 (80–95)	13.4 (5.8)	0.30 (0.03)	0.77 (0.01)	
Moderate	No Barrier	**Pop**	**8394 (11.9)**	**4271 (2545–11915)**	**21.2 (9.7)**	**0.79 (0.01)**	**0.84 (0.01)**	0.002
	Barrier	**Pop**	**8024 (14.1)**	**1936 (1214–4435)**	**19.5 (8.6)**	**0.78 (0.01)**	**0.84 (0.00)**	0.012
		Subpop	4012 (11.0)	1693 (1137–3169)	19.6 (8.8)	0.78 (0.01)	0.82 (0.01)	
			4010 (9.7)	1936 (1214–4435)	19.4 (8.6)	0.78 (0.01)	0.83 (0.01)	
	Culverts	**Pop**	**8024 (14.1)**	**1936 (1214–4435)**	**19.5 (8.6)**	**0.78 (0.01)**	**0.84 (0.00)**	0.003
		Subpop	4012 (11.0)	1693 (1137–3169)	19.6 (8.8)	0.78 (0.01)	0.82 (0.01)	
			4008 (9.5)	1683 (1100–3381)	20.7 (9.4)	0.79 (0.01)	0.83 (0.01)	
High	No Barrier	**Pop**	**12077 (16.2)**	**16271 (4774-∞)**	**22.1 (10.1)**	**0.81 (0.01)**	**0.84 (0.00)**	0.001
	Barrier	**Pop**	**11531 (19.7)**	**1216 (989–1550)**	**20.5 (9.2)**	**0.80 (0.01)**	**0.84 (0.00)**	0.008
		Subpop	5764 (14.2)	1525 (1014–2906)	20.7 (9.3)	0.80 (0.01)	0.83 (0.00)	
			5765 (14.9)	1814 (1160–3898)	20.5 (9.2)	0.80 (0.01)	0.83 (0.01)	
	Culverts	**Pop**	**11540 (22.7)**	**2334 (1329–8150)**	**21.6 (9.8)**	**0.81 (0.00)**	**0.84 (0.00)**	0.002
		Subpop	5777 (17.3)	3299 (1623–141173)	21.7 (9.9)	0.81 (0.01)	0.84 (0.00)	
			5761 (14.7)	2334 (1329–8150)	21.6 (9.8)	0.81 (0.01)	0.84 (0.00)	

Tortoise cell density was low (3/km^2^), moderate (14), or high (24). Resistance surfaces had no barrier, an impassable barrier, or barrier with culverts. Total number of individuals (*N*) and statistics are reported **in bold** at the population level (Pop) and for subpopulations (Subpop): (*Ne*) effective population size; (*Ar*) allelic richness; (*H*_*o*_) mean observed and (*H*_*e*_) expected heterozygosity; (*F*_*ST*_) pairwise genetic differentiation. Reported at generation 200 (approximately 3400 years). Initial simulation values are reported in Table 4.

**Fig 3:**
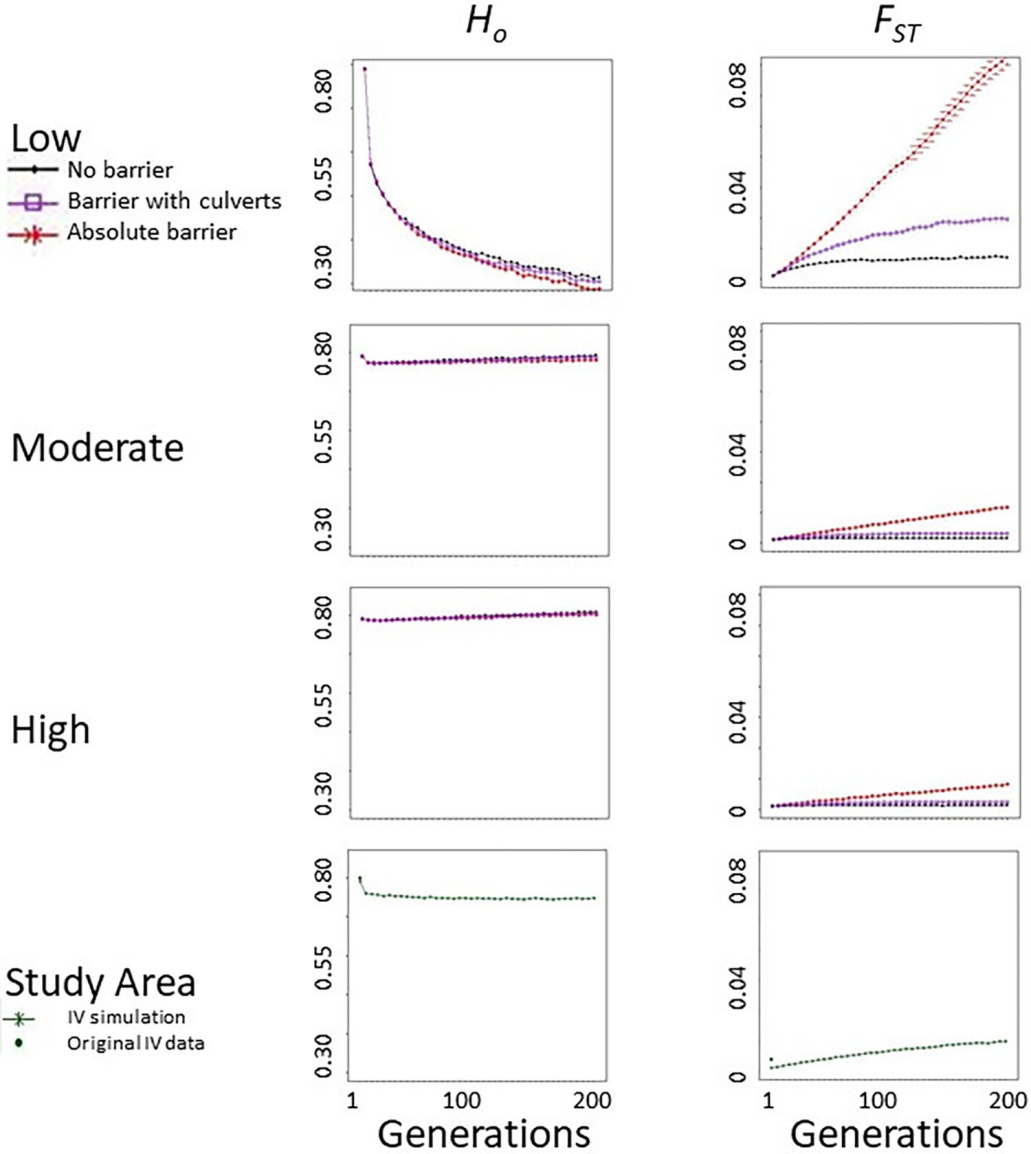
Predicted heterozygosity (*Ho*) and pairwise genetic differentiation (*F*_*ST*_) over time. Individuals were grouped relative to a linear barrier in three hypothetical landscapes (no barrier, absolute barrier, barrier with culverts) at three population densities (low, moderate, high) and a real world study area in the Ivanpah Valley (IV) along the Nevada/California border. In all hypothetical landscapes *F*_*ST*_ values for the scenario with culverts were intermediate, indicating that gene flow increased relative to an absolute barrier. At low densities, changes in *Ho* and *F*_*ST*_ likely reflected small population sizes that were increasingly isolated over time. Current estimates for IV likely indicate the time lag in detection, with *Ho* higher than predicted due to the scale and recentness of habitat loss and *F*_*ST*_ representing an existing signal of fragmentation from the interstate.

Initially genetic diversity (*Ar*, *H*_*o*_ and *H*_*e*_) was comparable among low, moderate, and high density simulations (Table 4 and Fig 3). At moderate population densities, heterozygosity did not differ over time with the no barrier resistance surface (*p* > 0.05, *df* = 19) and *Ar* had a *p*-value = 0.05. Genetic diversity remained stable with moderate population density between the no barrier and culvert scenarios (*Ar* and *H*_*o*_ *p* > 0.05, *df* = 19). Between the no barrier and barrier scenarios heterozygosity remained stable (*p* > 0.05, *df* = 19), but *Ar* decreased (*p* = 5 × 10^−6^, *df* = 19). The high population density simulations saw a significant increase in *Ar* (*p* = 7 × 10^−4^, *df* = 19). We found a significant decline in *Ar* between the no barrier and barrier simulations (*p* = 6 × 10^−4^, *df* = 19), but there were no differences between the no barrier and culvert scenarios (*p* > 0.05, *df* = 19). Heterozygosity also remained stable at high densities in the no barrier, absolute barrier, and barrier with culvert scenarios (*p* > 0.05, *df* = 19). However, at low population densities, genetic diversity dropped with the no barrier resistance surface (*Ar p* = 4 × 10^−11^ and *H*_*o*_ *p* = 3 × 10^−15^, *df* = 19). There were no significant differences in heterozygosity between the barrier scenarios (*p* > 0.05, *df* = 19), but *Ar* did decrease significantly (*p* = 4 × 10^−8^). When evaluated by subpopulation *Ar* showed a decreasing trend in the culvert and barrier simulations relative to the no barrier simulation at all densities (Table 5).

Initially, *F*_*ST*_ was low (< 0.002) in all simulations, indicating potentially high levels of genetic connectivity. No barrier landscapes displayed stability through time regardless of population density scenario. Our simulation results predicted increases in *F*_*ST*_ over time in absolute barrier simulations at all population densities. However, at low density, genetic differences were predicted to increase by an order of magnitude from no barrier to absolute barrier simulations (Fig 3). Low density simulations also displayed higher *F*_*ST*_ values in all landscape scenarios compared with moderate and high densities. Genetic differentiation for the barrier with culverts fell between the no barrier and absolute barrier landscapes at all simulated densities (Table 5).

Population genetic structure was initially absent in all simulated landscapes at all population densities. Over time the no resistance landscape revealed a pattern of IBD at all densities as the result of individual movement abilities. This conformed to expectations in natural populations. However, geographic population structure began emerging in simulations of low density (low density *K* = 3; moderate *K* = 1; high *K* = 1). We found that the absolute barrier created isolated populations regardless of simulated density (low, moderate, and high density *K* = 2). While the addition of connectivity culverts along the barrier did not alter the number of genetic clusters, admixture increased (low, moderate, and high density *K* = 2), indicating gene flow. We were able to detect population genetic structure earlier in time in low density simulations likely because genetic drift increased sensitivity.

Spatial genetic patterns of isolation were strongest in low population density simulations, and weakest with high density. By generation five a genetic signal of fragmentation was developing in low population density simulations with a linear barrier to dispersal (Mantel test *p* = 0.59 and *r* < -0.01, Moran’s I *p* < 0.01). Culverts also produced a signal of fragmentation at low density by the fifth generation; however, it was weaker than the absolute barrier simulation and allowed for some genetic connectivity (Mantel test *p* = 0.66 and *r* = -0.01, Moran’s I *p* = 0.03). By generation 40 isolation was emergent on the no barrier surface in the low density simulation (Mantel test *p* = 0.08 and *r* = 0.03, Moran’s I *p* < 0.01). The absolute barrier resulted in a signal of genetic fragmentation in moderate (Mantel test *p* 0.22 and *r* = 0.01, Moran’s I *p* < 0.01) and high density simulations (Mantel test and Moran’s I *p* < 0.01, Mantel test *r* = 0.04). Culverts also produced a signal of fragmentation by generation 40 at moderate density (Mantel test *p* = 0.33 and *r* = 0.01, Moran’s I *p* < 0.01). By generation 200 isolation developed at moderate density (Mantel test *p* = 0.55 and *r* < 0.01, Moran’s I *p* < 0.01), but was not apparent at high density (Mantel test *p* = 0.50 and *r* < 0.01, Moran’s I *p* = 0.06) on the no barrier surface. At high densities fragmentation was found with culverts by generation 200 (Mantel test *p* = 0.31 and *r* = 0.01, Moran’s I *p* < 0.01).

### Ivanpah Valley simulation

In the Ivanpah Valley, anthropogenic disturbance decreased the number of tortoises by 12% in the first five generations and by 15% by generation 40. Population census size remained stable thereafter. At the landscape level, *Ne* decreased 69% by generation five relative to the initial simulated subsample, 82% by generation 40, and 96% by generation 200 (Table 6). Subpopulation *Ne* followed similar patterns, decreasing 38–61% by generation 40 and 65–89% by generation 200 relative to generation five.

**Table 6:** Ivanpah Valley simulation results over time.

Time		Level	*N* (SD)	*Ne* (CI)	*Ar* (SD)	*H*_*o*_ (*SD*)	*H*_*e*_ (*SD*)	*F*_*ST*_
Generation	5	**Pop**	**6171 (26.8)**	**2138 (1574–3209)**	**19.1 (8.3)**	**0.76 (0.01)**	**0.82 (0.00)**	0.001
		Subpop	3023 (17.9)	1514 (1010–2866)	19.3 (8.5)	0.76 (0.01)	0.81 (0.00)	
			3146 (17.2)	1333 (925–2283)	19.1 (8.3)	0.77 (0.01)	0.82 (0.00)	
	40	**Pop**	**5995 (22.7)**	**1267 (1028–1623)**	**19.1 (8.3)**	**0.75 (0.01)**	**0.82 (0.00)**	0.004
		Subpop	2905 (18.2)	935 (711–1331)	19.4 (8.6)	0.75 (0.01)	0.82 (0.00)	
			3088 (14.4)	523 (438–640)	19.0 (8.2)	0.75 (0.01)	0.82 (0.00)	
	200	**Pop**	**5984 (22.2)**	**304 (281–329)**	**19.0 (8.3)**	**0.75 (0.01)**	**0.83 (0.01)**	0.010
		Subpop	2898 (16.6)	164 (152–178)	19.2 (8.4)	0.75 (0.01)	0.82 (0.01)	
			3084 (14.0)	470 (397–567)	18.9 (8.2)	0.74 (0.01)	0.82 (0.01)	

Total number of individuals (*N*) and statistics are reported **in bold** at the population level (Pop) and for each subpopulation (Subpop): (*Ne*) effective population size; (*Ar*) allelic richness; (*H*_*o*_) mean observed and (*H*_*e*_) expected heterozygosity; (*F*_*ST*_) pairwise genetic differentiation. Reported at generations 5, 40, and 200. Original data and initial simulation values are reported in Table 4.

Despite heterogeneous population density in the Ivanpah Valley simulation, *Ar* most closely aligned with our moderate density absolute barrier simulation (19.1 and 19.5; respectively). It is likely that the current disturbance landscape surface fragmented populations similarly to the absolute barrier simulation. Observed heterozygosity decreased through time by 4% at generation five and 5% at generation 40. Following 40 generations *H*_*o*_ stabilized in the Ivanpah Valley with existing disturbance (Fig 3). Empirical estimates of genetic diversity in the Ivanpah Valley are higher than predicted [27], likely due to the relatively recent timescale of development and the lag time to detect changes in genetic patterns. However, within five tortoise generations, we predict a loss of genetic diversity relative to current estimates as a result of recent disturbance.

Predicted pairwise *F*_*ST*_ was measured on either side of a linear barrier (Interstate 15) to infer gene flow. Initially, pairwise *F*_*ST*_ in the Ivanpah Valley simulation was significantly lower than the current expectation (*p* = 0.02). By generation 40, pairwise *F*_*ST*_ doubled relative to the empirical estimate. By the final generation simulated, predicted *F*_*ST*_ was almost an order of magnitude higher than the empirical estimate, similar to that predicted at moderate density with an absolute barrier (Table 6).

**Fig 4:**
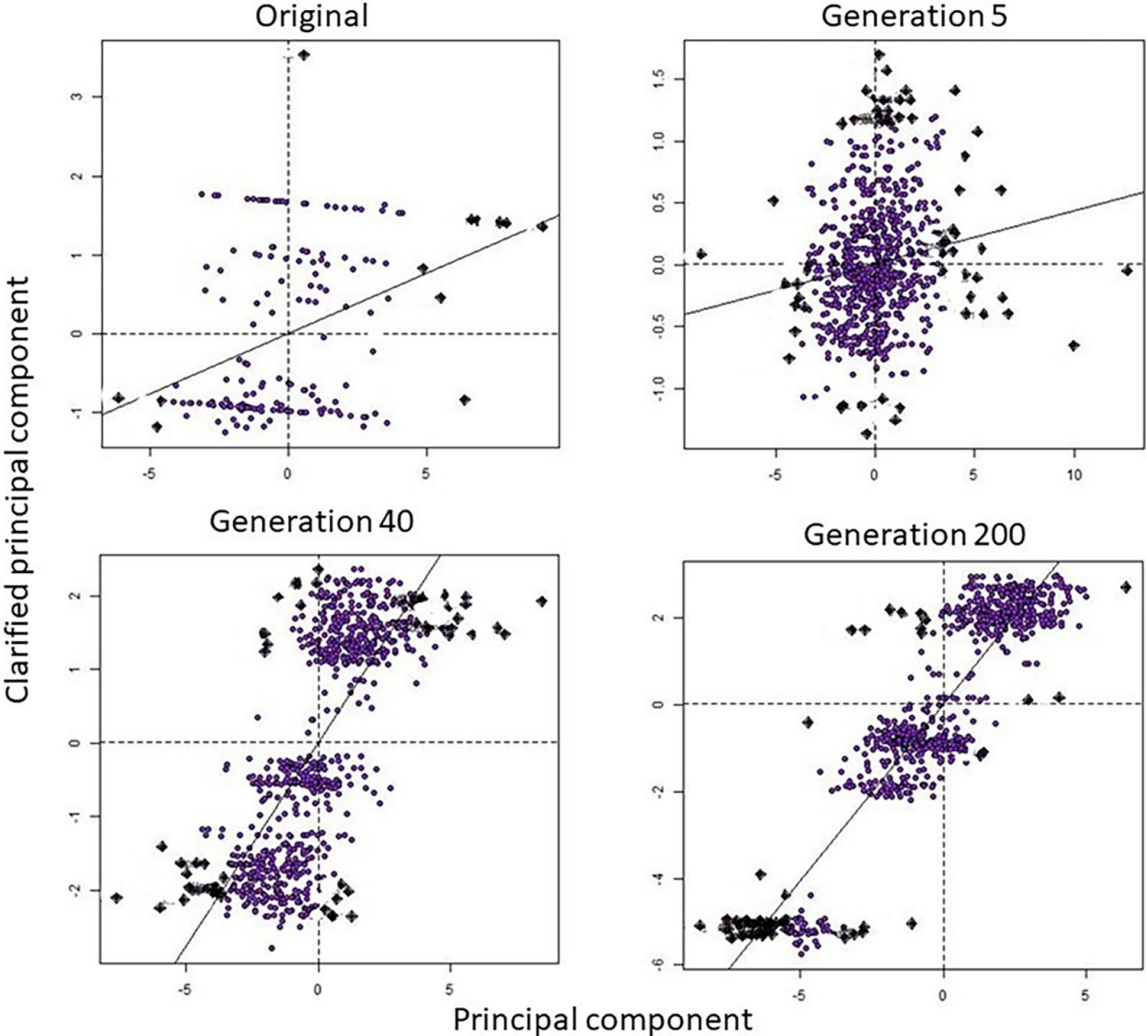
Predicted sPCA regression results. The original genetic data and simulation results from a real world study area in the Ivanpah Valley along the Nevada/California border forecasting an increased signal of fragmentation with current levels of disturbance over time. Results are shown at discrete generations (5, 40, 200). Points represent genotypes and spatial locations. A correlation between synthetic variables (principal components) indicates spatial autocorrelation and is shown by the linear regression line. Note differences in x and y axes.

Bayesian clustering analysis found an increased number of genetic clusters within the simulated area over time (*K* = 5), compared to the current single genetic cluster within Ivanpah Valley [27], indicating that populations became progressively isolated. We can currently detect weak genetic patterns on either side of the interstate in the Ivanpah Valley (Mantel test *p* = 0.15 and *r* = 0.04, Moran’s I *p* < 0.01). Our sPCA results from the Ivanpah Valley simulation indicated similar patterns of weak isolation from disturbance by generation five (Mantel test *p* = 0.84 and *r* = -0.02, Moran’s I *p* < 0.01) that became stronger with time (generation 40 Mantel test and Moran’s I *p* < 0.01, Mantel test *r* = 0.05). Linear regression of the results supports spatial population structure in all scenarios, with the slope becoming steeper and spatial isolation visibly apparent in the scatterplots as time increased, indicating greater isolation (Fig 4).

## Discussion

### Culverts maintain or improve gene flow

Understanding how barriers impact connectivity is crucial to conservation efforts [59]. In our Ivanpah Valley simulation, we found evidence of a reduction in gene flow on either side of the interstate by generation five (roughly 85 years). The loss of gene flow became more pronounced through time, resulting in a strong signal of isolation by generation 40 (roughly 680 years). This corresponds with empirical evidence from the Ivanpah Valley, where reduced gene flow has been associated with Interstate 15 and the Southern Pacific Railroad [27]. The results of our hypothetical landscape simulations provide additional support that barriers restrict gene flow. The genetic signature of fragmentation was strongest with an absolute barrier and increased through time. The addition of culverts restored some genetic connectivity, but did not entirely negate the genetic effects of a linear disturbance. These findings agree with simulation studies from a broad range of taxa where the addition of connectivity routes in fragmented landscapes is predicted to improve gene flow [12, 97, 98].

Tortoises have been documented using culverts and underpasses at variable rates, from no crossings at some locations up to 10 successful individual crossing events in a two-year period [99]. The number of possible crossing structures in our simulated culvert scenario underrepresented the number of existing potential crossing structures in the Ivanpah Valley. Every generation (roughly 17 years) our culvert scenarios allowed up to three individuals to use a crossing structure (a rate of roughly one crossing every 5.7 years per connectivity culvert). With three possible crossing locations in the 25 x 25 km landscape, a maximum of nine individuals could cross every generation (a rate of roughly one crossing every 1.9 years). Even with this conservative rate of culvert use, our results indicated that culverts under anthropogenic barriers, like roads and railways, are likely to improve connectivity for the desert tortoise.

### Population density influences connectivity

In simulations of habitat loss and fragmentation, Hand et al. [2] found evidence for strong non-linear critical thresholds in life history traits with relatively small losses in habitat and connectivity. In our moderate and high density simulations, population census size only decreased when a linear barrier was present due to the associated loss of habitat. Habitat loss was due to the wide footprint of the road itself (1 km^2^), which falls within estimated road effect zones that have found decreased tortoise abundance up to 4 km on either side of unfenced highways [29–31]. Habitat loss and degradation from current levels of disturbance in the Ivanpah Valley simulation produced fewer individuals and fewer breeding opportunities. The resulting genetic drift dramatically decreased effective population size relative to census size and is likely not significantly influenced by overlapping or non-overlapping generations [100]. We predicted losses of roughly 69% in effective population size and 12% in population census size by the fifth generation. Isolation among populations can develop within five to 10 generations in species with limited dispersal (such as desert tortoises) and with low population density [76].

Population declines associated with numerous unquantified threats have been documented in designated tortoise conservation areas with empirical estimates at or below 3/km^2^ [18]. Our results indicated that the greatest changes in connectivity are likely to occur within five generations and at low population density. In our low density simulations (3/km^2^), negative impacts occurred regardless of landscape surface, likely as the result of individuals moving without finding mates. Reduced adult reproductive rates and survivorship are both implicit drivers of tortoise population declines, although the causes of reduced reproductive output are unclear [54]. At maximum carrying capacity in low population density simulations, not all individuals in a cell could mate, which reduced population census size. This resulted in genetic drift, dramatic reductions in effective population sizes, and isolation. It is plausible that wild tortoises may be unable to find mates if densities fall too low; therefore, population density likely plays a principal role in maintaining connectivity. The present study is an initial examination showing a clear non-linear effect and a potential threshold between medium and low population densities under which connectivity is unsustainable.

### Model advantages and limitations

We implemented agent-based spatially explicit forward-in-time simulations of gene flow to track the number of individuals and their genotypes through time. Because gene flow and dispersal are population-level processes that occur over long time periods, our simulation models addressed large-scale spatial patterns over long time periods, rather than fine-scale dispersal.

The impacts of landscape change on genetic patterns are associated with a considerable lag time in detection [53, 101]. This can hinder detection following recent habitat disturbance. Therefore, better understanding of time to detection is important to interpreting empirical population genetic data following habitat disturbance. Our simulation framework allowed us to explore possible desert tortoise connectivity outcomes and time to detection. Over the 200 generation simulation period, tortoises will certainly experience additional changes in land use, climate, and vegetation to those presented here. However, our findings have practical implications now and can guide management actions towards the long-term persistence of the species.

Our simulation framework had several additional advantages for testing landscape genetic hypotheses. First, focusing on simplified landscapes allowed us to explore variations caused by a linear barrier and a barrier with culverts in the absence of any confounding influence from heterogeneous landscape structure. Second, we were able to evaluate differences arising from systematically varying population density, which impacted population census size. Third, we were able to examine current disturbance in the Ivanpah Valley. This added complexity, but also allowed for hypothesis testing related to the influence of multiple landscape features on gene flow.

The sampling grain or raster pixel size should ideally be smaller than an average home-range size and dispersal distance [101]. The mean home-range for desert tortoises is highly variable, but estimated to be roughly 1 km^2^ [25, 41]. Our 1 km^2^ grid cells represent areas in which individuals would likely make routine movements, capitalize on resources, and find mates. The cell size is also well below the expected dispersal distance for this species, which has been documented to be up to 13.6 km [63]. Using a sampling grain that is too large may smooth patterns, resulting in a decreased ability to detect the influence of landscape [101]. We were able to detect the influence of landscape, indicating that our grid cell size was appropriate. That said, using a smaller sampling grain, especially in areas of interest such as connectivity culverts along major roads, could reveal additional information relevant to these features.

A simplification to consider when interpreting our results is non-overlapping generations. The inclusion of overlapping generations in other platforms results in additional tradeoffs, such as constant population density, simulations of a single locus, or all individuals in a population assigned a single genotype (see: CDMetaPop [102]; CDPop [103]; MetaPopGen [104]). These simplifications would not have addressed our research questions. However, the effect of overlapping generations could be explored in future work to detect genetic signals of fragmentation in long lived organisms, like the desert tortoise. The use of overlapping generations may allow for more realistic movement parameters with more than one movement per generation.

In our simulations the destination cell was chosen randomly among possible cells, with breeding occurring when individuals occupied the same cell. Using a distribution function in future simulations to determine individual movements may influence dispersal probability and the rate of genetic exchange. In our low density simulations, mating opportunities were much reduced, reflected in very low effective population size estimates. This may be mitigated to some extent in real tortoise populations by social structure, such as shared burrows and mating behavior [78]. We assumed survivorship and breeding success to be equal for individuals. Building on this work by incorporating risk factors associated with age and habitat along with more realistic movement and reproductive behavior may provide additional information on reproduction and survival.

### Management considerations

The threat of habitat loss and fragmentation is likely to intensify in the foreseeable future [105]. A precipitous drop in the survival-habitat relationship indicates that as a population approaches this threshold, additional small losses of habitat have a large impact on the probability of survival [106]. Although this threshold is still unknown for the desert tortoise, habitat loss from development is substantial [107] and the number of individuals is declining [17–21]. Efforts to recover disturbed Mojave Desert lands may enhance success by increasing habitat amount, but recovery can be slow [108]. Ensuring adequate habitat is available by restricting anthropogenic disturbance to already disturbed areas, then restoring habitat where it has been lost and populations are declining may be an effective recovery strategy and has been proposed as part of a sustained management effort [109].

Roadside fencing reduces tortoise mortalities and may allow animals to reclaim depauperate habitat [32]. Retrofitting existing culverts and tortoise fencing to allow animals access to culverts is a feasible option to optimize safe passage and improve connectivity for tortoises [26, 28]. Future management practices that include optimal design and construction of crossing structures could further connectivity efforts in fragmented landscapes [110].

A unified network of conservation areas and connectivity routes at variable spatial scales, ranging from specific culverts across linear barriers to range-wide, may approximate natural patterns and maintain connectivity [111, 112]. Design and enactment of such a network is challenging, but would have long-term benefits for the persistence of the tortoise and the preservation of the Mojave Desert ecosystem. Existing research can serve to guide conservation and connectivity efforts. For example, Gray et al. [113] found strong evidence that tortoise movement is associated with increased vegetation cover. Therefore, areas with predicted high habitat quality and vegetation cover could be prioritized for protection. Management actions to increase vegetation cover in areas targeted for maintaining connectivity may also be effective.

Extinction risk may be underestimated if genetic factors are not considered in conservation strategies [114]. Changes in genetic diversity, differentiation, and structure may indicate more severe effects associated with population decline [6, 13, 115, 116]. We found estimates of population census size and effective population size yielded telling trends in five, or fewer, generations. Effective population size could be a useful metric to include in tortoise population monitoring efforts, along with continued population density monitoring. Incorporating genetic monitoring into management practices prior to disturbance, then periodically thereafter (i.e. every generation following disturbance), would allow patterns to be tracked through time.

## Supporting information

S1 Table Supplemental simulation methods. Initialization of state variables in simulations.
(DOCX)
