# Supplementary material for: Move it or lose it: Predicted effects of culverts and population density on Mojave desert tortoise (Gopherus agassizii) connectivity
Source: PLoS One. 2023 Sep 28;18(9):e0286820. doi: 10.1371/journal.pone.0286820 (PMC10538755; doi:10.1371/journal.pone.0286820)
Supplement: S1 Table — Initialization of state variables in simulations. (DOCX) [file pone.0286820.s001.docx]

**S1 Table: Supplemental Simulation Methods.**

|  | Description | Value | Justification/Reference |
| --- | --- | --- | --- |
| Population Dynamics | Logistic model growth rate | 0.48 | 1, 2 |
|  | Mortality | Logistic model | Allow for carrying capacity and growth rate |
|  | Mating distance | Within 1 km^2^ cell | 3, 4 |
|  | Sex ratio | 1:1 | 5 |
|  | Dispersal Probability | 0.5 | Hromada pers comm |
|  | Max dispersal distance | 10 cells, when possible | 6 |
|  | Individual location | Initially everywhere | Defined by landscape |
|  | Cell location of individuals | Cell centroids | Model default |
|  | Group classification | Based on linear feature | Allow for comparison |
| Genetics | Original genetic data | *Ivanpah Valley genetic cluster | 7, 8 |
|  | Microsatellite loci | 20 | 7, 8 |
|  | Mutation rate | 0.0005 | 9, 10, 11 |
| Simulation | Repetitions per sim | 30 | Allow for stochasticity |
|  | Generations per sim | 200 | Allow for lag time |

Initialization of state variables in simulations. *The plot locations within the Ivanpah Valley genetic cluster are: ISEGSNorth, ISEGSSouth, Nipton, Sheep, SilverState, Southpah.

**REFERENCES**

1. U.S. Fish and Wildlife Service. Desert tortoise (Mojave population) recovery plan. U.S. Fish and Wildlife Service, Portland, Oregon. 1994.

2. Medica PA, Nussear KE, Esque TC, Saethre MB. Long-term growth of desert tortoises (*Gopherus agassizii*) in a southern Nevada population. J Herpetol. 2012; 46(2): 213-220.

3. Berish JE, Medica PA. Home range and movements of North American tortoises. In: Rostal DC, McCoy ED, Mushinsky HR, editors. Biology and conservation of North American tortoises. John Hopkins University Press; 2014. pp 96-101.

4. Hromada SJ, Esque TC, Vandergast AG, Dutcher KE, Mitchell CI, Gray ME, et al. Using movement to inform conservation corridor design for Mojave desert tortoise. Mov Ecol. 2020; 8(38): 1-18.

5. Rostal DC, Wibbels T. Embryonic development, hatching success, and temperature dependent sex determination in North American tortoises. In: Rostal DC, McCoy ED, Mushinsky HR, editors. Biology and conservation of North American tortoises. John Hopkins University Press; 2014. pp 46-52.

6. Hromada SJ. The genes must flow: using movement ecology to understand connectivity of Mojave desert tortoise (*Gopherus agassizii*) populations in altered landscapes. Doctoral Dissertation, The University of Nevada, Reno. 2022.

7. Dutcher KE, Vandergast AG, Esque TC, Mitelberg A, Matocq MD, Heaton JS, et al. Genes in space: what Mojave desert tortoise genetics can tell us about landscape connectivity. Conserv Genet. 2020; 21(2): 289-303.

8. Dutcher KE, Vandergast AG, Esque TC, Mitelberg A, Nussear KE. Microsatellite genotypes for desert tortoise (*Gopherus agassizii*) in Ivanpah Valley (2015-2017): U.S. Geological Survey data release. 2020; Available from: htps://doi.org/10.5066/P90LIQRI.

9. Dileo MF, Rouse JD, Davila JA, Lougheed SC. The influence of landscape on gene flow in the eastern massasauga rattlesnake (*Sistrurus c. catenatus*): insight from computer simulations. Mol Ecol. 2013; 22(17): 4483-4498.

10. Estoup A, Angers B. Microsatellites and minisatellites for molecular ecology: theoretical and empirical considerations. In: Carvalho G, editor. Advances in molecular ecology. IOS Press Amsterdam; 1998. pp 55-86.

11. Landguth EL, Cushman SA, Schwartz MK, McKelvey KS, Murphy M, Luikart G. Quantifying the lag time to detect barriers in landscape genetics. Mol Ecol. 2010; 19(19): 4179-4191.
